# The strategy of laparoscopic surgery for asymptomatic antenatally diagnosed choledochal cyst

**DOI:** 10.1186/s12893-023-02095-3

**Published:** 2023-07-18

**Authors:** Xuepeng Zhang, Jianli Jin, Tong Qiu, Jiangyuan Zhou, Guowei Che, Yi Ji, Zhicheng Xu

**Affiliations:** 1 Department of Pediatric Surgery, West China Hospital, Sichuan University, #37 Guo- Xue-Xiang, Chengdu, 610041 China; 2 Department of Critical Care Medicine, West China Hospital, Sichuan University, Chengdu, 610041 China; 3 West China School of Nursing/West China Hospital, Sichuan University, Chengdu, 610041 Sichuan China; 4 Department of Thoracic Surgery, West China Hospital, Sichuan University, Chengdu, 610041 China

**Keywords:** Choledochal cyst, Prenatal diagnosis, Laparoscopy, Neonate, Small infant

## Abstract

**Background:**

The aim was to investigate the characteristics, surgical management, and outcomes of asymptomatic patients with antenatally diagnosed choledochal cysts (ADCCs) and to justify the strategy of laparoscopic surgery (LS) for them in our hospital.

**Methods:**

We developed our LS strategy for asymptomatic ADCCs. Patients with asymptomatic ADCCs who underwent LS or open surgery (OS) during January 2010 and January 2020 were included. Patients with recorded symptomatic ADCCs were exclude. All data of group LS and group OS were statistically compared and analyzed.

**Results:**

Twenty-five patients received LS and 18 patients received OS were included. There was no significant difference in baseline characteristics between the groups. A total of 65.1% of biliary sludge formation was detected preoperatively by ultrasonography (US) (72.0% in group LS, 55.6% in group OS, *P* = 0.26). Compared to the OS group, the LS group had a longer operative time (313.4 ± 27.2 vs. 154.0 ± 11.9 min, *P* = 0.02), shorter postoperative fasting period (3.1 ± 1.2 vs. 6.2 ± 2.3 days, *P* = 0.03), and shorter postoperative hospital stay (5.1 ± 1.9 vs. 9.2 ± 1.1 days, *P* = 0.03). The incidence of late complications, such as reflux cholangitis, adhesive intestinal obstruction, and biliary enteric anastomotic stricture with stone formation, was not significantly different between the two groups. The liver function and liver stiffness of all patients in the two groups were normal.

**Conclusions:**

Based on the strategy for asymptomatic ADCCs in our hospital, the perioperative safety and midterm follow-up results after LS were satisfactory and comparable to those after OS.

## Introduction

With the widespread use of routine maternal ultrasonography (US), an increasing number of antenatally diagnosed choledochal cysts (ADCCs) have been diagnosed. Of them, approximately 1/3 to 1/2 of patients are asymptomatic soon after birth, and the incidence of prenatal diagnosis increases with time [1, 2]. As a special entity of infantile choledochal cysts (CCs), ADCCs are known as a stenotic distal bile duct or blind-ending cyst, and cholestatic jaundice, liver fibrosis and cirrhosis start from an early stage of life [3]. Dewbury reported that liver cirrhosis could be confirmed as early as 10 days of age [4], and biliary sludge formation was detected by US as early as in the first 2 weeks of life [5]. However, if the surgical intervention is timely and effective, liver fibrosis and cirrhosis can be prevented without any sequelae.

Conventionally, because of the technical difficulties and the risks of anesthesia during the neonatal period, the traditional open surgery (OS) for ADCCs is postponed for 3–6 months. According to the Japanese clinical guidelines for pancreaticobiliary maljunction, symptomatic ADCCs should be operated on in the presence of symptoms, and elective surgery at approximately 3–6 months of age is recommended for asymptomatic ADCCs to avoid anastomotic complications [6]. With advancements in minimum invasive surgery (MIS), a growing number of infantile CCs have been resected under laparoscopy in neonates or small infants. For symptomatic ADCCs, the policy of laparoscopic surgery (LS) involvement is the same as that of traditional OS. However, for asymptomatic ADCCs, the optimal strategy for LS, especially the timing of the operation, is still controversial [3, 7–10].

## Materials and methods

Since January 2010, we have developed our LS strategy for asymptomatic ADCCs as follows: (1) The timing of surgery was scheduled when the patient’s age was > 2 months or weight was > 4 kg. (2) When clinical manifestations occur in patients waiting for elective operation, such as jaundice, acholic stool or vomiting, the LS procedure should be carried out immediately regardless of the patient’s age and weight. (3) The intraoperative cholangiogram (IOC) and intrahepatic biliary radicals (IHB) procedures were advocated for all patients. (4) Laparoscopic ductoplasty was not performed in hepaticojejunostomy when the diameter of the common hepatic duct (CHD) was narrowed. To validate this strategy, a retrospective study of asymptomatic ADCCs was conducted in our hospital.

This study was conducted according to Declaration of Helsinki, and was approved by the Ethics Committee of West China Hospital. The retrospective research was designed based on the medical in-hospital and outpatient charts of ADCCs between January 2010 and January 2020. Of them, the records of symptomatic ADCCs were excluded, and only those of asymptomatic ADCCs were enrolled, including those who received urgent resections during the waiting period. Preoperative US, computed tomography (CT), magnetic resonance cholangiopancreatography (MRCP), IOC, IHB, and the management of total cyst excision and Roux-en-Y hepaticojejunostomy were performed in all patients. Hepatocellular injury is defined as elevation of aminotransferase to more than two times the upper limit of the normal range. The histologic degree of hepatic fibrosis was scored by the new Inuyama classification [11]: grade 0, no fibrosis; grade 1, fibrosis localized in the portal area; grade 2, bridging formation of fibrosis to the neighboring portal area; grade 3, widened bridging fibrosis; and grade 4, pseudolobule formation, indicative of cirrhosis.

The patients underwent LS by authors were assigned to group LS, while others underwent OS by another surgical team were assigned to group OS. The patients’ data, including gestational age at diagnosis, sex, age and weight at surgery, diameter of CHD, type and maximum diameter of cyst, degree of hepatic fibrosis, operative time, intraoperative blood loss, postoperative fasting period and hospital stay, and intra- and postoperative complications, were all obtained using medical records. A follow-up timeline was set at 1, 3, 6, and 12 months postoperatively and once a year thereafter, when physical examinations, abdominal ultrasonographic studies, and blood liver function tests were taken at each visit. All data of group LS and group OS were statistically compared and analyzed.

### Laparoscopic procedure

Under general anesthesia, an approximately 1.0 cm horizonal incision was made in the center of the umbilical region. A 12 mm trocar for a 5 mm 30° laparoscope was placed using the Hasson technique, and a pneumoperitoneum of 8–12 mmHg was maintained with a flow rate of 3–6 L/min. Two additional 5 mm trocars were inserted at the right and left upper abdomen. The IOC was performed after exteriorization of the gallbladder via the right trocar incision in all patients. The gallbladder was then returned to the peritoneal cavity, and pneumoperitoneum was re-established using the parameters described above. The gallbladder and round ligament were suspended by percutaneous transperitoneal threads to fully expose the hepatic hilum. The cyst was completely resected, proximal to the CHD and distal to the pancreatic duct. Then, the ligament of Treitz was identified, and the jejunum 15 cm distal to the ligament was exteriorized through the umbilical port site. A 30–40 cm long Roux-en-Y limb was fashioned and then returned into the abdomen. After re-establishing the pneumoperitoneum, the Roux loop was delivered to the hilum via a retro-colic path. One layer of end-to-side hepaticojejunostomy was performed using interrupted one layer of 5–0 absorbable sutures. Finally, the liver biopsy was taken, the gallbladder was dissected from the liver, and the CC and gallbladder were removed through the umbilical incision. A drain was placed near the anastomotic site.

### Statistics

The software used for statistical calculation was IBM SPSS 22.0 for Windows 10.0 (SPSS, Inc., Chicago, IL, USA). Demographic comparisons were performed using Student’s t test or the nonparametric Mann–Whitney U test for continuous variables, where appropriate, and Fisher’s exact test or the chi-squared test was used for categorical variables. A P value < 0.05 was considered statistically significant.

## Results

Fifty-four patients with ADCCs received definitive resections between January 2010 and January 2020. Of them, 11 patients with symptomatic ADCCs were excluded. Finally, 43 patients with asymptomatic ADCCs were included in this study. US and CT were performed in all patients. Preoperative MRCP was carried out in the 15 patients, and the pancreaticobiliary junction could be identified in 8 of them. There were 34 patients with type I and 9 patients with type IVa in this study. Of the 43 patients, 25 patients underwent LS, while 18 patients underwent OS. There was no significant difference in age, sex, or weight between the two groups. The ratio of type I/IVa was 20/5 in the LS group and 14/4 in the OS group (*P* = 0.84). The incidence of biliary sludge formation detected by US preoperatively was 72.0% in the LS group and 55.6% in the OS group (*P* = 0.26). The median diameter of the CHD was similar in the two groups (3.5 ± 1.2 vs. 3.3 ± 0.9 mm, *P* = 0.74). The maximum diameter of cysts in the LS group was similar to that in the OS group (53.9 ± 4.3 vs. 52.3 ± 4.7 mm, *P* = 0.35) (Table 1).

**Table 1:** The comparison of preoperative data between two groups

	LS N = 25	OS N = 18	P value
Gestational age of diagnosis, weeks	23.9 ± 6.8	24.1 ± 8.2	0.43
Age at surgery, days	68.7 ± 14.6	65.3 ± 15.8	0.10
Male, n (%)	6 (24.0%)	6 (33.3%)	0.44
Weight, Kg	4.5 ± 2.9	4.3 ± 2.6	0.11
Type of cyst, n (%)			0.84
Type I Type IVa	20 (80.0%) 5 (20.0%)	14 (77.8%) 4 (22.2%)	
Biliary sludge formation, n (%)	18 (72.0%)	10 (55.6%)	0.26
Diameter of CHD, mm	3.5 ± 1.2	3.3 ± 0.9	0.74
Maximum diameter of cyst, mm	53.9 ± 4.3	52.3 ± 4.7	0.35

LS, laparoscopic surgery; OS, open surgery; CHD, common hepatic duct

During the waiting period for scheduled operations, 14 patients underwent prompt definite excisions; 9 of them (5 of type I, 4 of type IVa) received LS, while 5 (1 of type I, 4 of IVa) received OS. There were 9 patients with type IVa in this study, 8 of whom (88.9%) received urgent operations. Among the reasons for emergent surgery, 10 patients (71.4%) had jaundice with acholic stools, 2 patients (14.3%) had vomiting, and 2 patients (14.3%) had jaundice without other clinical symptoms. Biliary sludge formations in the cysts were preoperatively found by US in all patients.

There were no conversions to OS in group LS. No cases of intraoperative complications occurred in either group. The intraoperative blood loss was 17.5 ± 6.1 ml in the LS group, which was not significantly different from 20.3 ± 4.8 ml (*P* = 0.38) in the OS group. Compared to the OS group, the LS group had a longer operative time (313.4 ± 27.2 vs. 154.0 ± 11.9 min, *P* = 0.02), shorter postoperative fasting period (3.1 ± 1.2 vs. 6.2 ± 2.3 days, *P* = 0.03), and shorter postoperative hospital stay (5.1 ± 1.9 vs. 9.2 ± 1.1 days, *P* = 0.03). Postoperative early complications occurred in one patient in the LS group who had an omentum hernia from the trocar wound on the right upper abdominal wall when the drain was removed at 5 days. The omentum was reduced to the peritoneal cavity, and the abdominal wall wound was sutured again. The patient was discharged 2 days after surgery. Another early complication was seen in one patient in the OS group who had wound infection with partial dehiscence at 7 days. The wound was debrided and repaired, and the patient was discharged 5 days after surgery. The incidence of postoperative early complications was not significantly different between the two groups (Table 2).

**Table 2:** The comparison of intra and postoperative data between two groups

	Group LS N = 25	Group OS N = 18	*P* value
Operation time, min	313.4 ± 27.2	154.0 ± 11.9	0.02
Intraoperative blood loss, ml	17.5 ± 6.1	20.3 ± 4.8	0.38
Postoperative fasting period, days	3.1 ± 1.2	6.2 ± 2.3	0.03
Postoperative hospital stays, days	5.1 ± 1.9	9.2 ± 1.1	0.03
Intraoperative complications, n (%)	0	0	1.0
Early postoperative complication, n (%)	1 (4.0%)^*^	1 (5.6%)^#^	0.81

LS, laparoscopic surgery; OS, open surgery;

^*^ Herniation of the omentum;

^#^ Wound infection with partial dehiscence

The results of liver biopsy showed that all patients in both groups had different degrees of hepatic fibrosis. There were 19 cases of grade I, 5 cases of grade II and 1 case of grade III, while there were 13 cases of grade I, 4 cases of grade II, and 1 case of grade III in the OS group (*P* = 0.95) (Table 3). No cases of grade IV fibrosis were found within either group. The rate of inflammatory cell infiltration was similar in the two groups (12.0% vs. 11.1%, *P* = 0.93). There were 2 cases of hepatocellular injury in the LS group, which was not significantly different from 1 case in the OS group (8.0% vs. 5.6%, *P* = 0.76).

**Table 3:** The comparison of liver pathologic results between two groups LS, laparoscopic surgery; OS, open surgery

	Group LS N = 25	Group OS N = 18	*P* value
Inflammatory cell infiltration, n (%)	3 (12.0%)	2 (11.1%)	0.93
Hepatocellular injury, n (%)	2 (8.0%)	1 (5.6%)	0.76
Hepatic fibrosis, n (%)			0.95
Fibrosis I	19 (76.0%)	13 (72.2%)	
Fibrosis II	5 (20.0%)	4 (16.0%)	
Fibrosis III	1 (4.0%)	1 (5.6%)	
Fibrosis IV (cirrhosis)	0	0	

After the mean time of follow-up (67.8 ± 34.6 months after LS; 71.2 ± 31.3 months after OS; *P* = 0.63), the values of liver function and liver stiffness were normal in all patients in both groups. The ratio of reflux cholangitis after LS was similar to the ratio in the LS group (4.0% vs. 5.6%. *P* = 0.76). There were no cases of adhesive intestinal obstruction or biliary enteric anastomotic stricture with intrahepatic biliary stone formation after LS, but one case of adhesive intestinal obstruction (*P* = 0.23) and one case of biliary enteric anastomotic stricture with stone formation (*P* = 0.23) required surgery 2 years and 5 years after OS, respectively (Table 4).

**Table 4:** The comparison of follow-up results between two groups

	Group LS n = 25	Group OS n = 18	*P* value
Time of follow-up, months	67.8 ± 34.6	71.2 ± 31.3	0.63
Adhesive intestinal obstruction, n (%)	0 (0.0%)	1 (5.6%)	0.23
Anastomotic stricture with stones, n (%)	0 (0.0%)	1 (5.6%)	0.23
Reflux cholangitis, n (%)	1 (4.0%)	1 (5.6%)	0.76
The value of liver stiffness, Kpa	5.3 ± 0.8	5.1 ± 0.5	0.85

LS, laparoscopic surgery; OS, open surgery

## Discussion

Since the first report in 1995, the LS procedure has been used in congenital CCs for more than 27 years and has become a mature and mainstream option. Under laparoscopy, the view of the portal vein, hepatic artery and other deep anatomical structures are clearly amplified so that dissection and anastomosis can be meticulously performed without damaging the surrounding key structures. However, the LS procedure in neonates and small infants with limited working space has technical challenges and may have an impact on perioperative safety and postoperative efficacy. Based on the comprehension of obstructive biliary pathology deteriorating over time and the experiences of MIS accumulated in diseases of neonates and small infants, the strategy of LS for asymptomatic ADCCs has been established since January 2010 in our hospital.

Each patient was suggested for completion of IOC and IHB in our hospital, which may not only discern infantile CCs from cystic biliary atresia (BA) but also display the morphology of intra- and extrahepatic bile ducts. Occasionally, the aberrant hepatic duct can also be identified by intraoperative cholangiogram [12–14]. In this study, different levels of liver fibrosis were shown in all patients (grade I in 19, grade II in 5 and grade III in 1) in group LS, when the mean age and weight at operation were 66.1 ± 13.2 days and 4.5 ± 2.9 kg, respectively, which has been corroborated in the literature [3, 4, 7]. Although the hepatic fibrosis of infantile ADCCs is reported more commonly, it has been basically considered a reversible pathology with no influence on the postoperative course [15, 16]. However, once the treatment is delayed, liver fibrosis inevitably evolves into liver cirrhosis with an incidence of 2.1–11.8% [3, 7, 17], which is regarded as an irreversible disorder, especially when the stage of cirrhosis is Child‒Pugh class B or C [18].

In addition, it is particularly concerning that the progression of liver fibrosis may be related to type IVa infantile CCs, whose postoperative course in advanced cases may be unsatisfactory. There were 9 patients with type IVa asymptomatic ADCCs (5 in group LS, 4 in group OS) in this study. Eight patients (88.9%) received prompt resections (4 for LS, 4 for OS) during the waiting period due to the emergence of clinical symptoms, such as acholic stools, obstructive jaundice, and vomiting. All of them were found to have biliary sludge formation with more severe liver fibrosis (grade II,7; grade III,2), which may indicate various degrees of biliary obstruction or even spontaneous perforation of the biliary system [19]. Hori et al. listed 11 cases with congenital CCs seeking liver transplantation, 2 of whom were 0.4-year-old pediatric patients with type IVa CC [9]. Another 3-month-old girl with type IVa CC was reported requiring liver transplantation due to liver cirrhosis, despite the emergent decompression of the bile duct had been performed before [10]. Therefore, early surgical intervention is especially warranted for type IVa infantile CCs [9, 20].

Based on the extensive experiences of MIS for esophageal atresia, duodenal atresia, BA, and children CCs, the timing of LS for asymptomatic ADCCs in our hospital was customized as when the patient’s age was > 2 months or weight was > 4 kg, which was similar to the LS was safe and feasible in infants < 3 months with asymptomatic ADCCs [21]. However, Diao et al. recommended neonatal LS for asymptomatic ADCCs because the incidence of hepatic fibrosis was significantly increased when surgery was performed after 1 month of age [22]. On the other hand, van den Eijnden et al. suggested delaying operation until the age of 6 months or a weight of 6 kg in asymptomatic ADCCs because all postoperative short-term and long-term complications occurred in patients weighing < 5.6 kg at the time of LS [23]. In general, the timing of LS for asymptomatic ADCCs should be established and weighed individually between perioperative safety and postoperative efficacy. In our study, the hepatic pathological results mentioned above assumed a delay of treatment; however, after a mean time of 67.8 ± 34.6 months of follow-up, all patients in group LS had normal values of liver function and liver stiffness (5.3 ± 0.8 KPa) by ultrasound elastography (USE). Nonetheless, 5 patients (20%) after LS were still complicated with liver fibrosis, resulting in prolonged liver dysfunction for more than 2 years, which was consistent with the observations from some references [8, 24, 25].

The management of laparoscopic hepaticojejunostomy within the limited intraperitoneal space of neonates or small infants was highly demanding, in which a slow learning curve was observed [26, 27]. The operative time of LS was significantly longer than that of OS, indicating that there was still a certain learning curve to be passed in our hospital. In addition, the stricture of CHD was reported to be one of the features of infantile CCs [28]. Although ductoplasty was described as an option to facilitate hepaticojejunostomy in the case of small CHD < 5 mm [29, 30], we strongly support the suggestions that performing laparoscopic ductoplasty in a friable CHD is not a safe method in a neonate or small infant [22]. Furthermore, we did not notice any stricture of CHD by intraoperative cholangiogram in our study. The diameter of CHD in group LS was 3.5 ± 1.2 mm, which was sufficient to enable laparoscopic hepaticojejunostomy without ductoplasty. Of them with a CHD diameter > 3.0 mm, only one-layer interrupted sutures around the anastomotic stoma were accomplished with a margin distance > 5 mm. Of them with a CHD diameter < 3.0 mm, especially in patients who received prompt LS during the waiting period, 4 stitches of one layer around the anastomotic stoma were sewn with a margin distance of 3 ~ 5 mm. If it was difficult to handle, the stitches of anastomosis could be preset first and then knotted in turn. During the follow-up in our study, there were no cases of anastomotic leakage, stricture, or biliary stone formation after LS, which was better than the 6–40% anastomotic complications reported [31–34]. For giant CCs > 10 cm in diameter, bile liquids should be drained from the filled sac to facilitate the release of the working space after IOC. In addition, by applying a series of transabdominal suture retractions, especially sequential suture retractions along the giant cyst dissection direction, the distal end of the common bile duct could be fully exposed in conventional 3-port LS.

According to the policy above run in our hospital, LS procedures for neonates and small infants with asymptomatic ADCCs were carried out without intraoperative events in our study. The postoperative fasting period and hospital stay after LS were significantly shorter than those after OS. The postoperative early complications were only seen in one patient with hernia of the omentum in the LS group (1/25, 4.0%), which was not significantly different from that of one patient with wound infection in the OS group (1/18, 5.6%). Postoperative late complications were observed in one patient with reflux cholangitis (1/25, 4.0%) after LS, which was not different from one case of reflux cholangitis (1/18, 5.6%), one case of adhesive intestinal obstruction (1/18, 5.6%), and one case of biliary anastomotic construction with stone formation after OS (1/18, 5.6%). Fortunately, all patients in both groups had normal values of liver function and liver hardness by USE without any signs of liver cirrhosis after follow-up.

Nonetheless, there were some limitations associated with this study. The analyses were conducted retrospectively in a small number of patients, which could cause type II errors. Second, the length of follow-up was not long enough to draw more convincing conclusions. In addition, during follow-up, the development of liver fibrosis should be evaluated by percutaneous liver biopsy, which is more accurate and reliable than US.

In conclusion, the strategy of LS for asymptomatic ADCCs adopted in our study was effective with satisfactory perioperative safety and better midterm results, which was comparable to that of OS. Considering the above achievements, the current limit of body weight and age for LS in asymptomatic ADCCs may be further reduced as the surgeon’s laparoscopic skill improves.

## Data Availability

The datasets used and/or analyzed during the current study are available from the corresponding author on reasonable request.

## Abbreviations

ADCC: Antenatally diagnosed choledochal cyst
BA: Biliary atresia
CC: Choledochal cyst
CHD: Common hepatic duct
CT: Computed tomography
IHB: Intrahepatic biliary radicals
IOC: Intraoperative cholangiogram
LS: Laparoscopic surgery
MIS: Minimum invasive surgery
MRCP: Magnetic resonance cholangiopancreatography
OS: Open surgery
US: Ultrasonography
USE: Ultrasound elastography

## References

[1] Matsumoto M, Urushihara N, Fukumoto K, Yamoto M, Miyake H, Nakajima H (2016). Laparoscopic management for prenatally diagnosed choledochal cysts. Surg Today.

[2] Urushihara N, Fukumoto K, Yamoto M, Miyake H, Takahashi T, Nomura A, Sekioka A, Yamada Y, Nakaya K (2018). Characteristics, management, and outcomes of congenital biliary dilatation in neonates and early infants: a 20-year, single-institution study. J Hepato-Biliary-Pancreat Sci.

[3] Suita S, Shono K, Kinugasa Y, Kubota M, Matsuo S (1999). Influence of age on the presentation and outcome of choledochal cyst. J Pediatr Surg.

[4] Dewbury KC, Aluwihare AP, Birch SJ, Freeman NV (1980). Prenatal ultrasound demonstration of a choledochal cyst. Br J Radiol.

[5] O’NEILL JJPs: Choledochal cyst. Pediatric surgery. 2006:1620–1634.

[6] Kamisawa T, Ando H, Suyama M, Shimada M, Morine Y, Shimada H (2012). Japanese clinical practice guidelines for pancreaticobiliary maljunction. J Gastroenterol.

[7] Chen CJ (2003). Clinical and operative findings of choledochal cysts in neonates and infants differ from those in older children. Asian J Surg.

[8] Tsai MS, Lin WH, Hsu WM, Lai HS, Lee PH, Chen WJ (2008). Clinicopathological feature and surgical outcome of choledochal cyst in different age groups: the implication of surgical timing. J Gastrointest surgery: official J Soc Surg Aliment Tract.

[9] Hori T, Oike F, Ogura Y, Ogawa K, Hata K, Yonekawa Y, Ueda M, Sakamoto S, Kasahara M, Egawa H (2010). Liver transplantation for congenital biliary dilatation: a single-center experience. Dig Surg.

[10] Diao M, Li L, Cheng W (2011). Is it necessary to ligate distal common bile duct stumps after excising choledochal cysts?. Pediatr Surg Int.

[11] Ichida F, Tsuji T, Omata M, Ichida T, Inoue K, Kamimura T, Yamada G, Hino K, Yokosuka O, Suzuki H (1996). New Inuyama classification; new criteria for histological assessment of chronic hepatitis. Int Hepatol Commun.

[12] Takahashi T, Shimotakahara A, Takahashi T, Lee KD, Lane GJ, Okazaki T, Yamataka A (2008). Choledochal cyst associated with an accessory hepatic duct identified by intra-operative endoscopy: case report and literature review. Pediatr Surg Int.

[13] Goor DA, Ebert PA (1972). Anomalies of the biliary tree. Report of a repair of an accessory bile duct and review of the literature. Archives of surgery (Chicago Ill: 1960).

[14] Momiyama T, Souda S, Yoshikawa Y, Kuratani T, Toda K, Koma M (1996). Injury to a duplicated cystic duct during laparoscopic cholecystectomy. Surg laparoscopy endoscopy.

[15] Ishimaru T, Kitano Y, Uchida H, Kawashima H, Gotoh C, Satoh K, Yoshida M, Kishimoto H, Iwanaka T (2010). Histopathologic improvement in biliary cirrhosis after definitive surgery for choledochal cyst. J Pediatr Surg.

[16] Jackson CC, Wu Y, Chenren S, Somme S, Chwals WJ, Liu DC (2002). Bile decompression in children with histopathological evidence of pre-existing liver cirrhosis. Am Surg.

[17] Chang MH, Wang TH, Chen CC, Hung WT (1986). Congenital bile duct dilatation in children. J Pediatr Surg.

[18] Serpaggi J, Carnot F, Nalpas B, Canioni D, Guéchot J, Lebray P, Vallet-Pichard A, Fontaine H, Bedossa P, Pol S (2006). Direct and indirect evidence for the reversibility of cirrhosis. Hum Pathol.

[19] Howell CG, Templeton JM, Weiner S, Glassman M, Betts JM, Witzleben CL (1983). Antenatal diagnosis and early surgery for choledochal cyst. J Pediatr Surg.

[20] Gong ZH, Xiao X, Chen L (2007). Hepatic fibrosis with choledochal cyst in infants and children - an immunohistochemical assessment. Eur J Pediatr surgery: official J Austrian Association Pediatr Surg [et al] = Zeitschrift fur Kinderchirurgie.

[21] Chan KW, Lee KH, Tsui SY, Mou JW, Tam YH (2016). Laparoscopic management of antenatally detected choledochal cyst: a 10-year review. Surg Endosc.

[22] Diao M, Li L, Cheng W (2012). Timing of surgery for prenatally diagnosed asymptomatic choledochal cysts: a prospective randomized study. J Pediatr Surg.

[23] van den Eijnden MHA, de Kleine RH, de Blaauw I, Peeters P, Koot BGP, Oomen MWN, Sloots CEJ, van Gemert WG, van der Zee DC, van Heurn LWE (2017). The timing of surgery of antenatally diagnosed choledochal malformations: a descriptive analysis of a 26-year nationwide cohort. J Pediatr Surg.

[24] Fujishiro J, Urita Y, Shinkai T, Gotoh C, Hoshino N, Ono K, Komuro H (2011). Clinical characteristics of liver fibrosis in patients with choledochal cysts. J Pediatr Surg.

[25] Hasegawa T, Kimura T, Ihara Y, Fukuzawa M (2006). Histological classification of liver fibrosis and its impact on the postoperative clinical course of patients with congenital dilatation of the bile duct. Surg Today.

[26] Diao M, Li L, Cheng W (2011). Laparoscopic versus open Roux-en-Y hepatojejunostomy for children with choledochal cysts: intermediate-term follow-up results. Surg Endosc.

[27] Wang B, Feng Q, Mao JX, Liu L, Wong KK (2012). Early experience with laparoscopic excision of choledochal cyst in 41 children. J Pediatr Surg.

[28] Diao M, Li L, Cheng W (2011). Congenital biliary dilatation may consist of 2 disease entities. J Pediatr Surg.

[29] Burnweit CA, Birken GA, Heiss K (1996). The management of choledochal cysts in the newborn. Pediatr Surg Int.

[30] Lee SC, Kim HY, Jung SE, Park KW, Kim WK (2006). Is excision of a choledochal cyst in the neonatal period necessary?. J Pediatr Surg.

[31] Todani T, Watanabe Y, Urushihara N, Noda T, Morotomi Y (1995). Biliary complications after excisional procedure for choledochal cyst. J Pediatr Surg.

[32] Kim JH, Choi TY, Han JH, Yoo BM, Kim JH, Hong J, Kim MW, Kim WH (2008). Risk factors of postoperative anastomotic stricture after excision of choledochal cysts with hepaticojejunostomy. J Gastrointest surgery: official J Soc Surg Aliment Tract.

[33] Cho MJ, Hwang S, Lee YJ, Kim KH, Ahn CS, Moon DB, Lee SK, Kim MH, Lee SS, Park DH (2011). Surgical experience of 204 cases of adult choledochal cyst disease over 14 years. World J Surg.

[34] Kim JW, Moon SH, Park DH, Lee SS, Seo DW, Kim MH, Lee SK (2010). Course of choledochal cysts according to the type of treatment. Scand J Gastroenterol.

